# Celiac Artery Compression Syndrome as an Uncommon Cause of Intractable Postprandial Abdominal Pain: A Case Report

**DOI:** 10.7759/cureus.32434

**Published:** 2022-12-12

**Authors:** Ali M Mahgoub, Ahmed H Abdelfattah, Hadeel Dawoud, Ahmed N Elkot

**Affiliations:** 1 Gastroenterology and Hepatology Unit, Department of Internal Medicine, Mansoura University, Specialized Internal Medicine Hospital, Mansoura, EGY; 2 Department of Internal Medicine, University of Kentucky College of Medicine, Lexington, USA; 3 Department of Critical Care Medicine, Mansoura University, Mansoura, EGY

**Keywords:** cacs, celiac artery compression syndrome, unintentional weight loss, postprandial abdominal pain, median arcuate ligament release, the median arcuate ligament compresses the celiac trunk

## Abstract

Celiac artery compression syndrome (CACS) is an uncommon and poorly understood condition. Compression of the celiac artery by the median arcuate ligament causes intractable postprandial abdominal pain, weight loss, vomiting, and nausea. We present a case of a 68-year-old male who suffered recurrent severe episodes of postprandial abdominal pain associated with occasional nausea, vomiting, and elevated blood pressure. The diagnostic workup was significant for celiac artery compression on computed tomography angiography. Diagnosis of CACS was made after the exclusion of the other possible pathologies, and the patient was referred to the surgical team for further management for median arcuate ligament release on an elective basis.

## Introduction

Celiac artery compression syndrome (CACS) is a rare condition caused by the compression of the celiac artery by the median arcuate ligament, which is a fibrous band of the diaphragm. It is also known as median arcuate ligament syndrome (MALS). CACS is a rare cause of postprandial abdominal pain with an incidence of two out of 100,000 patients [1]. The clinical presentation usually is postprandial abdominal pain, nausea, vomiting, nutritional deficiencies, and weight loss [1]. It can be easily confused with chronic mesenteric ischemia due to the similar presentation [2]. Diagnosis can be confirmed by computed tomography angiography (CTA), and the treatment is done by different surgical approaches. The median arcuate ligament release has demonstrated the best results [2].

## Case presentation

We present a case of a 68-year-old male with a medical history significant for diabetes and hypertension. He presented with recurrent severe abdominal pain for about two weeks. The pain was tearing in nature, epigastric, and periumbilical. The pain did not radiate or change with certain positions. It was aggravated with food intake and could last for several hours. It was associated with occasional nausea and vomiting. The patient reported that he used to have mild epigastric pain that was aggravated by meals for the past couple of years. There was no diarrhea, constipation, fever, changes in the urine or stool colors, or signs of gastrointestinal (GI) bleeding.

On presentation, he was hemodynamically stable, with elevated blood pressure during the pain attacks. Physical examination was significant for epigastric tenderness with normal bowel sounds. His laboratory findings are shown in Table 1.

**Table 1:** Laboratory findings

	Laboratory value	Reference range
Hemoglobin	12.7 mg/dl	12-17 mg/dl
Hematocrit (Hct)	41.9%	41-50%
White count	8.1 k/mm^3^	4.3-11 k/mm^3^
Platelets	197*10^3^/mm^3^	150-450*10^3^/mm^3^
Total bilirubin	1.1 mg/dl	0.5-1.2 mg/dl
Direct bilirubin	0-29 mg/dl	Up to 0.25 mg/dl
Indirect bilirubin	0.81 mg/dl	Up to 1.0 mg/dl
Aspartate aminotransferase (AST)	42.0 U/L	Up to 40 U/L
Alanine aminotransferase (ALT)	36.0 U/L	Up to 45 U/L
Prothrombin time (PT)	12.9 seconds	11-13 seconds
International normalized ratio (INR)	1.02	1.0-3.0
Serum creatinine	1.1 mg/dl	0.6-1.2 mg/dl
Potassium	3.9 mmol/L	3.5-5.5 mmol/L
Amylase	67.0 U/L	Up to 86
Lipase	51.0 U/L	13.00-60.00

**Figure 1:**
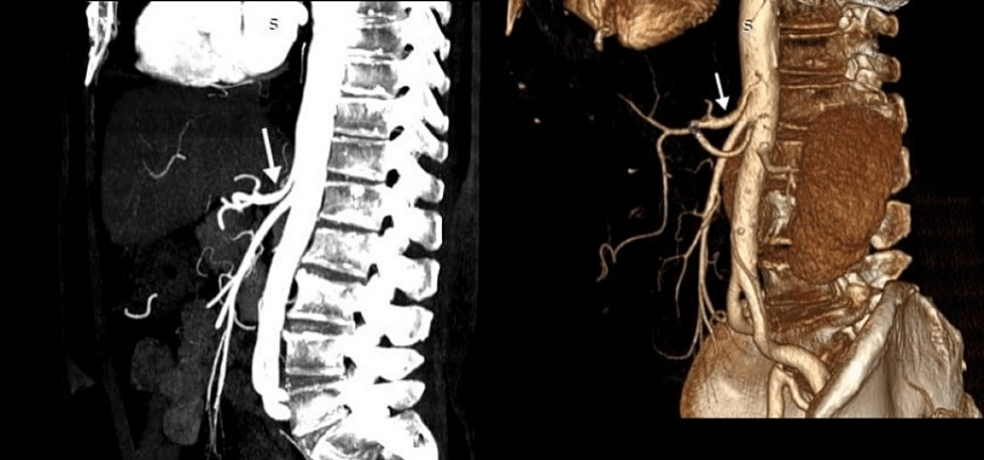
Computed tomography angiography of the abdomen and pelvis (sagittal view) with two white arrows showing the compression of the celiac artery

**Figure 2:**
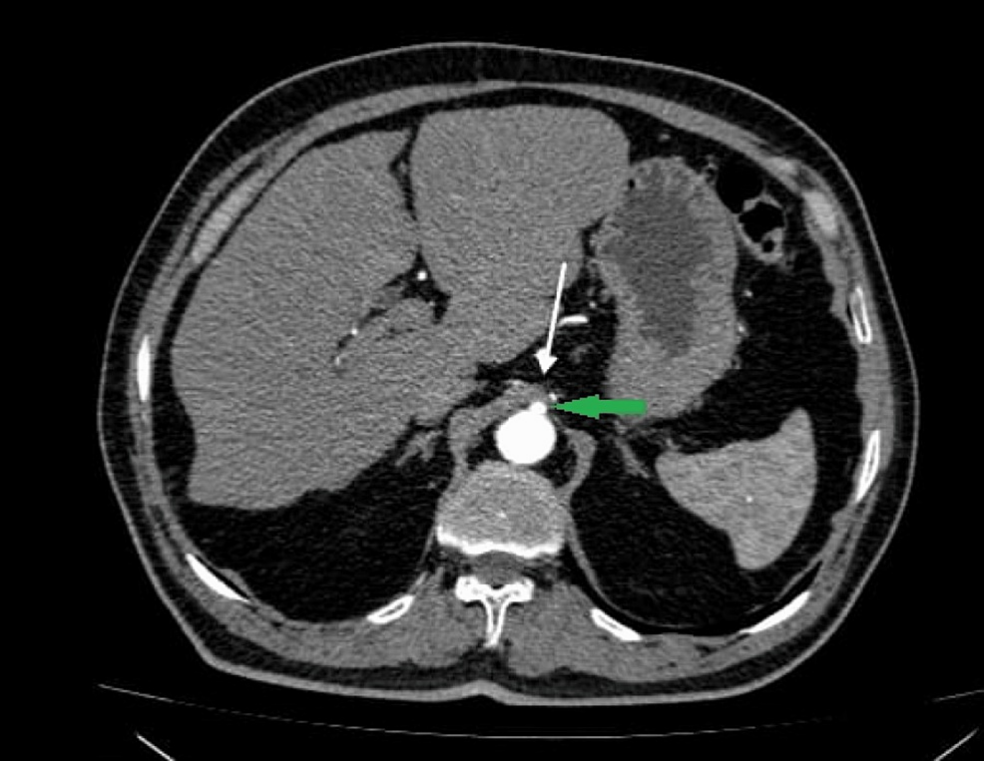
Computed tomography angiography of the abdomen (horizontal view) showing the median arcuate ligament (white arrow) compressing the origin of the celiac trunk (green arrow)

Electrocardiogram (EKG) showed normal sinus rhythm. Abdominal ultrasound was done, and it did not show significant findings. An esophagogastroduodenoscopy (EGD) was done and showed mild nonspecific gastritis. CTA was done with suspicion of chronic mesenteric ischemia, and it showed significant compression of the origin of the celiac trunk by the median arcuate ligament suggestive of MALS, with normal mesenteric circulation. There were no hepatic or biliary abnormalities (Figures 1, 2).

Diagnosis of CACS was made based on the clinical presentation and radiological findings. Other possible causes like acute pancreatitis, mesenteric ischemia, peptic ulcers, and symptomatic gallstones were excluded. Conservative management was started then the patient was referred to the surgical team for further management for median arcuate ligament release on an elective basis.

## Discussion

CACS is an uncommon condition caused by compression of the celiac trunk by the median arcuate ligament. It causes impaired blood supply to the GI tract [1]. The first time this condition was described was back in 1917 by Lipshutz. Forty-six years later, Harjola reported celiac artery compression in a female patient presenting with unexplained abdominal pain, and afterward, several case reports continued to shed the light on this rare condition, which led to the understanding of the role of celiac artery compression in causing ischemic intestinal pain. However, the fact that this condition only affects about two per 100,000 population makes it an overlooked diagnosis [3]. Gut ischemic pain can present with postprandial abdominal pain, nausea, vomiting, and weight loss [2]. Sometimes this pain can improve with inspiration or kyphotic posturing, as both can relieve the compression partially [2]. Not only do patients with CACS suffer from ischemic pain, but also some of these symptoms may be attributed to celiac ganglion compression leading to neuropathic pain [4].

CACS can be found incidentally in many asymptomatic patients showing radiographic signs of celiac artery compression [5]. CACS can mimic other abdominal pathologies like chronic mesenteric ischemia, symptomatic gall stones, or pancreatitis; therefore, it is diagnosed after excluding other possible pathologies [2,5]. It is essential to notice that only 40% of patients with CACS present with gut angina, likely due to the development of sufficient collateral blood supply to the affected region [6], which makes the diagnosis challenging. Patients often undergo extensive workups for other causes of abdominal pain and may have unnecessary procedures, imaging, and surgeries prior to the diagnosis being made [6].

Imaging modalities like CTA or abdominal duplex ultrasound (US) with respiratory maneuvers can be used to make the diagnosis after the exclusion of other potential etiologies [6,7]. One of the radiological findings that can be detected in the duplex US is increased systolic velocities at the celiac artery origin, which can increase by expiration and normalize with inspiration [8]. CTA usually shows a characteristic focal narrowing with a hooked appearance in the celiac artery origin, which can be useful in differentiating CACS from other causes of celiac artery stenosis such as atherosclerosis [8].

Different surgical approaches have been discussed in the literature like open, laparoscopic, or robotic ligament release, celiac ganglionectomy, and celiac artery revascularization. Angioplasty has a limited role in CACS management since this intervention does not address the underlying extrinsic compression resulting in symptoms, although angioplasty with stenting may be used in refractory cases [9]. Surgical management should be done for symptomatic patients and asymptomatic patients with more than 50% stenosis of the celiac artery [2]. Median arcuate ligament release is the most successful approach with an 85% chance of postoperative symptom relief [10]. Other studies showed that the recurrence rate can be as high as 38% [11]. Symptomatic relief can be achieved in some cases with the celiac plexus neurolysis approach. Unfortunately, a minority of patients continue to experience symptoms following treatment in the absence of other underlying diseases [2].

## Conclusions

CACS is a rare condition with significant consequences. Affected patients may develop intolerable postprandial abdominal pain, causing severe malnutrition and weight loss. The results of surgical management are varied but usually cause significant symptom improvement. Unfortunately, recurrence can occur, and a minority of patients continue to have symptoms despite surgical treatment.
